# Gender Differences in COVID-19 Conspiracy Theory Beliefs

**DOI:** 10.1017/S1743923X20000409

**Published:** 2020-07-09

**Authors:** Erin C. Cassese, Christina E. Farhart, Joanne M. Miller

**Affiliations:** 1 University of Delaware; 2 Carleton College; 3 University of Delaware

**Keywords:** Gender gap, conspiracy beliefs, learned helplessness, COVID-19, public opinion

## Abstract

In this article, we evaluate gender differences in COVID-19 conspiracy theory beliefs. We find that women are significantly less likely than men to endorse COVID-19 conspiracy theories and that this gender difference cuts across party lines. Our analysis suggests that this gender gap is partially explained by two dispositional factors: learned helplessness and conspiratorial thinking. Our findings qualify past work on the antecedents of conspiracy theory beliefs, which does not uncover robust and significant gender differences. The results highlight the need for work in this area to better theorize about the significance of gender.

The COVID-19 pandemic has given rise to a number of conspiracy theories (CTs) about its origin, seriousness, and attempts to stop its spread. Research exploring the causes of these beliefs finds that Republicans, conservatives, people higher in personal uncertainty, and people who are predisposed to conspiratorial thinking and denialism are more likely to believe COVID-19 CTs than their counterparts (Miller 2020a, 2020b; Uscinski et al. 2020). In this article, we shine the spotlight on another potential correlate of COVID-19 CT beliefs—gender. Previous conspiracy theory scholarship has devoted little attention to gender (see Douglas et al. 2019 for a comprehensive review). Gender is typically treated as a control variable in multivariate models aimed at testing theories about other correlates of conspiratorial thinking; a review of this work suggests no consistent differences between men and women across a range of CT beliefs.^1^ Yet preliminary evidence suggests that men and women are experiencing the pandemic differently, both in terms of health outcomes and more broadly, which raises questions about the existence of gender differences in endorsement of COVID-19 CTs (Wenham, Smith, and Morgan 2020).

## ARE MEN AND WOMEN EQUALLY LIKELY TO ENDORSE COVID-19 CONSPIRACY THEORIES?

To investigate gender differences in COVID-19 CT beliefs, we surveyed 3,019 Americans between April 24 and April 28, 2020. Participants were recruited by Lucid Theorem. Lucid provides quota samples that are matched to U.S. Census Bureau benchmarks. (Coppock and McClellan 2019 compare data gathered via Lucid's platform and MTurk and conclude that Lucid is appropriate for social science research). We created a set of survey weights using a ranked weighting procedure based on the 2018 Current Population Survey (CPS) benchmarks for education, income, sex, race, and ethnicity to more closely approximate the U.S. population. Survey weights are applied in all of the analysis presented here (see Appendix A in the supplementary material online for a comparison of sample demographics with 2018 CPS benchmarks).

We measured perceptions of the 11 COVID-related CTs summarized in Table 1. The order of the CTs was randomized across respondents. Responses were coded from 1 to 4 (definitely not true; probably not true; probably true; definitely true); higher numbers equate to greater belief (see Appendix B for survey items). The top row associated with each CT in Table 1 reports the percentage of men and women in the full sample indicating that each COVID CT is “probably” or “definitely” true. Women are significantly less likely to endorse each of the 11 CTs than men. The average gender gap across CTs is 10.18 percentage points. On average, men endorse 4.43 and women endorse 3.31 COVID-19 CTs—this difference is statistically significant at the *p* < .001 level.

**Table 1.** Gender Differences in Endorsement of Conspiracy Theories

		Men	Women	*Diff.*
The virus is a biological weapon intentionally released by China.	All	52	45	7**
	Democrats	42	34	8*
	Republicans	63	60	3
The virus was accidentally released by China.	All	57	49	8***
	Democrats	47	41	6^+^
	Republicans	67	57	10**
The virus was accidentally released by the US.	All	23	15	8***
	Democrats	24	14	10***
	Republicans	24	15	9**
Scientists are exaggerating the seriousness to make President Trump look bad.	All	41	22	19***
	Democrats	26	14	12***
	Republicans	55	31	24***
The media are exaggerating the seriousness to make President Trump look bad.	All	53	34	19***
	Democrats	36	20	16***
	Republicans	70	52	18***
Democratic Governors are hoarding ventilators to make President Trump look bad.	All	43	29	14***
	Democrats	30	14	16***
	Republicans	58	48	10***
Democratic Governors are not distributing coronavirus tests to make President Trump look bad.	All	43	38	5*
	Democrats	30	22	8**
	Republicans	56	55	1
5G technology is causing the coronavirus to spread faster.	All	26	17	9***
	Democrats	24	16	8**
	Republicans	30	17	13***
The coronavirus isn't real.	All	10	6	4***
	Democrats	8	5	3^+^
	Republicans	13	6	7***
Former Microsoft CEO Bill Gates is creating a tracking device to be injected with the coronavirus vaccine.	All	51	42	9***
	Democrats	49	36	13***
	Republicans	55	49	6^+^
The coronavirus was intentionally created to reduce the world's population.	All	43	33	10***
	Democrats	41	29	12***
	Republicans	47	37	10**
Average Number of CTs Endorsed	All	4.43	3.31	1.12***
	Democrats	3.56	2.45	1.11***
	Republicans	5.38	4.28	1.10***

Entries are the percentage of men and women indicating each CT is “probably” or “definitely” true. Survey weights are applied. Significance tests are chi-squared tests, with the exception of the average number of CTs endorsed, which is an F-test. Partisan categories include independent-leaning partisans. +p < .10 *p < .05, **p < .01, ***p < .001

Given that women are significantly more likely to self-identify as Democrats (e.g., Barnes and Cassese 2017) and that Democrats are significantly less likely to endorse COVID-19 CTs than Republicans (Miller 2020b; Uscinski et al. 2020), it is possible that the gender gap in COVID-19 CT beliefs is driven by gender differences in partisanship. To explore this possibility, we also report gender differences in CT beliefs by party (see Table 1). The partisan categories include independent-leaning partisans. Consistent with Miller (2020b) and Uscinski et al. (2020), Republicans are more likely to endorse COVID-19 CTs than Democrats. However, party differences do not explain away the gender gap in CT beliefs. Among Democrats, there are statistically significant gender gaps for all 11 CTs; among Republicans, there are statistically significant gender gaps for nine of 11 CTs. The average gender gap among Democrats is 10.18 points, compared with 10.09 points among Republicans— thus, gender differences in COVID-19 CT beliefs are relatively robust and cut across party lines. These gender differences are notable, given that gender gaps in issue attitudes tend to be much smaller in magnitude than what we observe here (Barnes and Cassese 2017). These results are surprising given that past work does not find a consistent association between gender and CT beliefs.

## WHAT FACTORS ACCOUNT FOR THE GENDER GAP IN COVID-19 CT BELIEFS?

Our preliminary analysis suggests that the gender gap in CT beliefs does not stem from gender differences in partisanship. We turn back to the CT literature to identify other potential explanations that are both situational (i.e., related to the pandemic context) and dispositional in nature. On the situational side, epistemic needs for certainty and control give rise to CT beliefs (see Douglas et al. 2019 for a review). Consistent with this literature, Miller (2020b) finds that personal uncertainty (likely induced by the pandemic) is positively related to COVID-19 CT beliefs. In addition to situational inducements of uncertainty, people who are higher in learned helplessness (LH) are more likely to engage in conspiratorial thinking (Farhart, Miller, and Saunders 2020). LH refers to the attributions of helplessness that some individuals make when they perceive that they have little or no control over aversive events (e.g., Seligman 1972). Another dispositional factor that is positively related to CT beliefs (including endorsement of COVID-19 CTs; see Miller 2020b; Uscinski et al. 2020) is conspiratorial thinking—the tendency to view events as the product of a conspiracy (Uscinski and Parent 2014).

Given the strong associations between CT beliefs and uncertainty, learned helplessness, and conspiratorial thinking, we measured these three factors to explore whether they explain the gender gap in COVID-19 CT beliefs.

• *Personal uncertainty* was measured using a three-item, 4-point Likert scale that assessed how uncertain people felt about themselves, their place in the world, and their future (alpha = .87).
• *Learned helplessness* was measured using a five-item, 4-point agree/disagree scale validated by Farhart (2017) (alpha = .89).
• *Conspiratorial thinking* was measured using a four-item, 5-point agree/disagree scale validated by Uscinski et al. (2016) (alpha = .81).

Our dependent variable is a COVID-19 CT index consisting of the average of responses to the 11 CT beliefs listed in Table 1. Prior work has established that these items are monological (Miller 2020a), thus justifying the composite index (alpha = .86). All independent and control variables were recoded to range from 0 to 1.

Before turning to our main findings, it is instructive to explore gender differences in LH, personal uncertainty, and conspiratorial thinking. Past work on these topics does not find consistent gender differences; however, we do observe some here. In bivariate comparisons, men score significantly higher on learned helplessness (*F*[1,3016] = 28.25, *p* < .001) and conspiratorial thinking (*F*[1,3016] = 43.62, *p* < .001) compared to women. Men also score modestly higher on our measure of uncertainty, though the difference is not statistically significant (*F*[1,3016] = 2.52, *p* = .11).

To better understand the gender gap in CT beliefs, we estimated a series of ordinary least squares (OLS) regression models, adding LH, uncertainty, and conspiratorial thinking to the models in a stepwise fashion (along with a set of controls). We use seemingly unrelated regressions to compare the size of the coefficient for the variable “female” across models. A significant reduction in the size of the “female” coefficient when the factor (either LH, uncertainty, or conspiratorial thinking) is included in the model indicates that the gender gap is at least partially explained by that factor.

The results of the OLS models are presented in Table 2. Model I estimates the gender gap in COVID-19 CT beliefs controlling for demographic, personality, and attitudinal factors. The difference between men and women is statistically significant (*p* < .001), consistent with our observations from the bivariate relationships presented in Table 1. Model II includes dummy variables for partisanship, with Democrats serving as the baseline category. Party has a significant effect on CT beliefs, with both independents and especially Republicans endorsing more CTs. The coefficient size for gender is reduced slightly, from –.18 to –.16, though the difference is statistically significant (*F*[1,2573] = 6.53, *p* = .01). This result suggests party is only a modest contributor to the gender gap in CT beliefs, consistent with the bivariate results presented in Table 1, and much of the gender gap stems from other factors.

**Table 2.** Sources of the Gender Gap in Conspiracy Theory Beliefs

	I	II	III	IV	V	VI
	Controls Only	+ Party	+ Learned Helplessness	+ Uncertainty	+ Conspiratorial Thinking	All Covariates
Female	−.18***	−.16***	−.12***	−.16***	−.11***	−.09***
	(.03)	(.02)	(.02)	(.02)	(.02)	(.02)
Age	−.15**	−.13*	.01	−.04	−.09	−.02
	(.06)	(.06)	(.05)	(.06)	(.05)	(.05)
Education	.07	.05	.06	.04	.02	.02
	(.05)	(.05)	(.04)	(.04)	(.04)	(.04)
Income	−.02	−.03	−.02	−.03	−.02	−.02
	(.04)	(.04)	(.04)	(.04)	(.04)	(.04)
White	.01	−.08*	−.09**	−.09**	−.04	−.06
	(.03)	(.03)	(.03)	(.03)	(.03)	(.03)
Hispanic	.06	.06	.07	.05	.05	.05
	(.04)	(.04)	(.04)	(.04)	(.04)	(.04)
Religiosity	.39***	.32***	.28***	.31***	.22***	.21***
	(.04)	(.04)	(.04)	(.04)	(.04)	(.04)
Authoritarianism	−.04	−.06	−.04	−.04	−.08*	−.06
	(.04)	(.04)	(.04)	(.04)	(.04)	(.04)
Ideology	.32***	.08	.13*	.12*	.10	.12*
	(.05)	(.06)	(.05)	(.06)	(.05)	(.05)
Knowledge	−.79***	−.85***	−.71***	−.77***	−.64***	−.59***
	(.06)	(.05)	(.05)	(.06)	(.05)	(.05)
Trust in Government	.45***	.35***	.25***	.31***	.37***	.32***
	(.05)	(.05)	(.05)	(.05)	(.04)	(.05)
COVID Concern	−.27***	−.23***	−.30***	−.30***	−.26***	−.30***
	(.05)	(.05)	(.05)	(.05)	(.04)	(.04)
COVID News	.05	.10	.10	.08	.04	.05
	(.06)	(.06)	(.06)	(.06)	(.06)	(.06)
Independent		.11*	.10**	.11**	.13**	.12**
		(.04)	(.04)	(.04)	(.04)	(.04)
Republican		.38***	.36***	.37***	.35***	.35***
		(.03)	(.03)	(.03)	(.03)	(.03)
Learned Helplessness			.67***			.31***
			(.06)			(.06)
Uncertainty				.41*** (.05)		.08 (.06)
Conspiratorial Thinking					.97***	.82***
					(.05)	(.06)
Constant	2.32***	2.39***	2.03***	2.19***	1.77***	1.66***
	(.07)	(.07)	(.08)	(.08)	(.08)	(.08)
*N*	2574	2526	2526	2526	2526	2526
*R^2^*	.34	.39	.45	.42	.51	.52

Entries are OLS regression coefficients with standard errors in parentheses. Survey weights are applied.

+p < .10 *p <.05, **p < .01, ***p < .001

We next consider whether LH, uncertainty, and/or conspiratorial thinking account for some of the observed differences between men and women. As Models III–V show, learned helplessness, uncertainty, and conspiratorial thinking are all associated with higher levels of COVID-19 CT beliefs (consistent with past research). However, only two of these factors reduce the estimated effect of respondent gender, meaning they are implicated in the gender gap. Specifically, we find that inclusion of learned helplessness (see Model III) results in a significant reduction in the effect of gender on CT beliefs compared to Model II (*F*[1,2525] = 21.58, *p* < .001), as does the inclusion of conspiratorial thinking (see Model V; *F*[1,2525] = 26.13, *p* < .001). Whereas uncertainty has a main effect on CT beliefs, its effect is unrelated to gender (see Model IV; *F*[1,2525] = 0.87, *p* = .35). Model VI in Table 2 includes LH, uncertainty, and conspiratorial thinking, to provide a better sense of their simultaneous effect on CT beliefs. In this model, the effect of uncertainty goes to zero, whereas LH and especially conspiratorial thinking continue to have large and significant effects on COVID CT beliefs.

## CONCLUSION

In sum, we find that men are more likely to endorse COVID-19 CTs than women, and this gender gap is robust to model specifications that include a wide array of controls.^2^ Moreover, we find that the gender gap is not due solely to partisanship or to uncertainty (likely induced by the pandemic) but is partially explained by LH and conspiratorial thinking—two psychological dispositions on which men score significantly higher in our data. Although drawing strong causal conclusions about the mediational process at work is beyond the scope of our data, these results are consistent with the notion that gender has an influence on COVID-19 CT beliefs that works though dispositional factors.

Our findings depart from past literature on CTs, which finds mixed evidence of gender differences in CT beliefs. This work is a first step toward understanding gender differences in response to the COVID-19 pandemic. Public health research shows that women are more likely to engage in preventive health measures (e.g., wearing masks and social distancing) than men (Padilla 2020). While our data do not situate us to evaluate this difference in behavior, our analysis gestures at attitudinal factors which may underpin these self-protective behaviors. To the extent that engaging in these behaviors enables women to feel like they have more control over the threats posed by the pandemic, they may serve as a buffer against CT beliefs. A better understanding of how people grapple with helplessness and uncertainty—and the role that gender plays in this process—is important for understanding the antecedents of politically and socially consequential CT beliefs, as well as compliance with the recommendations of public health experts in this kind of high-risk pandemic context.

## Footnotes

1. For example, Miller, Saunders, and Farhart (2016) find no effect of gender on conservative or liberal CTs, and Farhart, Miller, and Saunders (2020) find no effect of gender on conspiratorial thinking. Women score higher on an index of conspiratorial thinking and an index of conservative CTs in one of Federico, Williams, and Vitriol's (2018) two studies but not in the other; there was no gender effect on nonideological CTs. The bivariate relationship between gender and CT beliefs is also mixed. Uscinski and Parent (2014) found no difference between men and women in conspiratorial thinking, and Freeman and Bentall (2017) found that men were significantly more likely to agree with the statement that “I am convinced there is a conspiracy behind many things in the world.”

2. Our results diverge from those of Uscinski et al. (2020), who find no effect of their gender control variable on two COVID-19 CT beliefs. Our model and Uscinski et al.'s (2020) are different in a variety of respects, making direct comparisons difficult. We look forward to future research that explores the conditions under which gender does and does not predict CT beliefs.

## Supplementary material

For supplementary material accompanying this paper visit https://doi.org/10.1017/S1743923X20000409. click here to view supplementary material

## References

[ref1] Barnes, Tiffany D., and Erin C. Cassese. 2017 “American Party Women: A Look at the Gender Gap within Parties.” Political Research Quarterly 70 (1): 127–41.

[ref2] Coppock, Alexander, and Oliver A. McClellan. 2019 “Validating the Demographic, Political, Psychological, and Experimental Results Obtained from a New Source of Online Survey Respondents.” Research & Politics 6 (1). 10.1177/2053168018822174.

[ref3] Douglas, Karen M., Joseph E. Uscinski, Robbie M. Sutton, Aleksandra Cichocka, Turkay Nefes, Chee Siang Ang, and Farzin Deravi. 2019 “Understanding Conspiracy Theories.” Advances in Political Psychology 40 (1): 3–35.

[ref4] Farhart, Christina E. 2017. “Look Who Is Disaffected Now: Political Causes and Consequences of Learned Helplessness in the U.S.” PhD diss., University of Minnesota Twin Cities.

[ref5] Farhart, Christina E., Joanne M. Miller, and Kyle L. Saunders. 2020 “Conspiracy Stress or Relief? Learned Helplessness and Conspiratorial Thinking” In The Politics of Truth, eds. Elizabeth Suhay and David Barker, 1–38. Oxford University Press.

[ref6] Federico, Christopher M., Allison L. Williams, and Joseph A. Vitriol. 2018 “The Role of System Identity Threat in Conspiracy Theory Endorsement.” European Journal of Social Psychology 48: 927–938. 10.1002/ejsp.2495.

[ref7] Freeman, Daniel, and Richard P. Bentall. 2017 “The Concomitants of Conspiracy Concerns.” Social Psychiatry and Psychiatric Epidemiology 52 (5): 595–604. 10.1007/s00127-017-1354-4. 28352955 PMC5423964

[ref8] Miller, Joanne M. 2020a “Do COVID-19 Conspiracy Theory Beliefs form a Monological Belief System?” Canadian Journal of Political Science/Revue canadienne de science politique. Published online May 21. 10.1017/S0008423920000517.

[ref9] Miller, Joanne M. 2020b “Psychological, Political, and Situational Factors Combine to Boost COVID-19 Conspiracy Theory Beliefs” Canadian Journal of Political Science/Revue canadienne de science politique. Published online June 11. 10.1017/S000842392000058X

[ref10] Miller, Joanne M., Kyle L. Saunders, and Christina E. Farhart. 2016 “Conspiracy Endorsement as Motivated Reasoning: The Moderating Roles of Political Knowledge and Trust.” American Journal of Political Science 60 (4): 824–44.

[ref11] Padilla, Mariel. 2020 “Who's Wearing a Mask? Women, Democrats and City Dwellers.” *New York Times*, June 2. https://www.nytimes.com/2020/06/02/health/coronavirus-face-masks-surveys.html (accessed July 10, 2020).

[ref12] Seligman, Martin E. P. 1972 “Learned Helplessness.” Annual Review of Medicine 23 (1): 407–12. 10.1146/annurev.me.23.020172.002203 4566487

[ref13] Uscinski, Joseph E., Adam M. Enders, Casey M. Klofstad, Michelle Seelig, John Funchion, Caleb Everett, Stephan Wuchty, Kamal Premaratne, and Manohar Murthi. 2020 “Why Do People Believe COVID-19 Conspiracy Theories?” Harvard Kennedy School Misinformation Review. 10.37016/mr-2020-015. PMC8345314 34368805

[ref14] Uscinski, Joseph E., Casey Klofstad, and Matthew D. Atkinson. 2016 “What Drives Conspiratorial Beliefs? The Role of Informational Cues and Predispositions.” Political Research Quarterly 69 (1): 57–71.

[ref15] Uscinski, Joseph E., and Joseph M. Parent. 2014 American Conspiracy Theories. Oxford: Oxford University Press.

[ref16] Wenham, Clare, Julia Smith, and Rosemary Morgan. 2020 “COVID-19: The Gendered Impacts of the Outbreak.” The Lancet 395(10227): 846–48. 10.1016/S0140-6736(20)30526-2 PMC7124625 32151325

