# Supplementary material for: Gender Differences in COVID-19 Conspiracy Theory Beliefs
Source: Politics & Gender. 2020 Jul 9:1–10. doi: 10.1017/S1743923X20000409 (PMC7588715; doi:10.1017/S1743923X20000409)
Supplement: Supplementary file 1 [file S1743923X20000409sup001.docx]

**Online Supplementary Materials for**

**Gender Differences in COVID-19 Conspiracy Theory Beliefs**

**Appendix A. Comparison of Raw and Weighted Lucid Data to Current Population Survey (U.S. Census 2018) Benchmarks**

|  |  |  |  |
| --- | --- | --- | --- |
|  | Raw Data | Weighted Data | CPS 2018 |
| Female | 53% | 51% | 51% |
| College Degree | 44% | 35% | 31% |
| Black/Non-Hispanic | 11% | 12% | 13% |
| White/Non-Hispanic | 69% | 61% | 62% |
| Hispanic | 11% | 15% | 18% |
| Age (mean) | 46 | 45 | 37 |
| Income (median) | $40-44,999 | $55-59,999 | $55-59,999 |

Note: Weights adjust for gender, education, race, ethnicity, and income.

**Appendix B. Question Wordings and Variable Coding**

**Conspiracy Theories**

All conspiracy theory questions were coded to range from 1-4, with higher numbers representing greater belief.

[BioChina] Some people believe that the coronavirus is actually a biological weapon that was released from a laboratory in China. Others do not believe this. What do you think?

The coronavirus is: definitely a Chinese biological weapon; probably a Chinese biological weapon; probably not a Chinese biological weapon; definitely not a Chinese biological weapon.

[AccChina] Some people believe that the coronavirus was accidentally released from a laboratory in China. Others do not believe this. What do you think? The coronavirus was: definitely accidentally released from a Chinese lab; probably accidentally released from a Chinese lab; probably not accidentally released from a Chinese lab; definitely not accidentally released from a Chinese lab.

[AccUS] Some people believe that the coronavirus was accidentally released from a laboratory in the United States. Others do not believe this. What do you think? The coronavirus was: definitely accidentally released from a U.S. lab; probably accidentally released from a U.S. lab;

probably not accidentally released from a U.S. lab; definitely not accidentally released from a U.S. lab.

[Scientists] Some people believe that fear-mongering scientists are intentionally exaggerating the seriousness of the coronavirus to make President Trump look bad. Others do not believe this. What do you think? Scientists are: definitely exaggerating the seriousness of the coronavirus; probably exaggerating the seriousness of the coronavirus; probably not exaggerating the seriousness of the coronavirus; definitely not exaggerating the seriousness of the coronavirus.

[Media] Some people believe that fear-mongering media outlets are intentionally exaggerating the seriousness of the coronavirus to make President Trump look bad. Others do not believe this. What do you think? Media outlets are: definitely exaggerating the seriousness of the coronavirus; probably exaggerating the seriousness of the coronavirus; probably not exaggerating the seriousness of the coronavirus; definitely not exaggerating the seriousness of the coronavirus.

[NotReal] Some people believe that the coronavirus isn't real, and that doctors and scientists are in on the elaborate hoax. Others do not believe this. What do you think? The coronavirus: definitely is not real; probably is not real; probably is real; definitely is real.

[Hoard] Some people believe that Democratic Governors are hoarding ventilators to make President Trump look bad. Others do not believe this. What do you think? Democratic Governors are: definitely hoarding ventilators; probably hoarding ventilators; probably not hoarding ventilators; definitely not hoarding ventilators.

[Tests] Some people believe that Democratic Governors are intentionally not distributing the coronavirus tests the federal government has given them to make President Trump look bad. Others do not believe this. What do you think? Democratic Governors are: definitely not distributing all the coronavirus tests they have; probably not distributing all the coronavirus tests they have; probably distributing all the coronavirus tests they have; definitely distributing all the coronavirus tests they have.

[Tech] Some people believe that 5G technology is causing the coronavirus to spread faster. Others do not believe this. What do you think? 5G technology is: definitely causing the coronavirus to spread faster; probably causing the coronavirus to spread faster; probably not causing the coronavirus to spread faster; definitely not causing the coronavirus to spread faster.

[Gates] Some people believe former Microsoft CEO Bill Gates is creating a tracking device to be injected with the coronavirus vaccine. Others do not believe this. What do you think? Bill Gates is: definitely creating a tracking device for the vaccine; probably creating a tracking device for the vaccine; probably not creating a tracking device for the vaccine; definitely not creating a tracking device for the vaccine.

[WorldPop] Some people believe that the coronavirus was intentionally created as a plot to reduce the world's population. Others do not believe this. What do you think? The coronavirus was: definitely created to reduce the world's population; probably created to reduce the world's population; probably not created to reduce the world's population; definitely not created to reduce the world's population.

[COVID-19 CT Index] An index was created by averaging responses to the 11 CTs described above. Responses could range from 1-4, with higher numbers representing greater belief in COVID-19 CTs.

**Female**

A dummy variable [Female] was created such that 0=male and 1=female.

**Age**

Respondent age ranged from 18-88 and was recoded to range from 0-1.

**Education**

Education was coded into 4 categories ranging from 0-1: less than high school, high school graduate, some college, and college graduate or more.

**Income**

Income was coded into quartiles ranging from 0-1: less than or equal to $19,999, $20,000-44,999, $45,000-79,999, and $80,000 or more

**White**

A dummy variable [White] was created such that 0=not White and 1=White.

**Hispanic**

A dummy variable [Hispanic] was created such that 0=not Hispanic and 1= Hispanic.

**Religiosity**

How would you classify your level of involvement with your religion or spirituality? Responses to this question were coded to range from 0-1, with higher numbers meaning more active. Response options were: very active, moderately active, neither active nor inactive, moderately inactive, very inactive.

**Ideology**

We hear a lot of talk these days about liberals and conservatives. Here is a seven-point scale on which the political views that people might hold are arranged from extremely liberal to extremely conservative. Where would you place yourself on this scale? Extremely liberal, liberal, slightly liberal, moderate; middle of the road, slightly conservative, conservative, extremely conservative. Responses were coded to range from 0-1 with higher numbers meaning more conservative.

**Political Knowledge**

Now we have a set of questions concerning politics and public figures. We want to see how much political information gets out to the public from television, newspapers and the like. Please don’t get any outside help (from the Internet, asking somebody, etc.) for these questions. We are also interested in how long it takes people to answer these questions, so we will be recording the time it takes you to answer each one.

Would you say that one of the parties is more conservative than the other at the national level? Republican Party, Democratic Party, neither party is more conservative than the other.

What job or political office is now held by John Roberts? Chair of the Democratic National Committee, Senate Majority Leader, Chief Justice of the Supreme Court.

Who is the current Speaker of the U.S. House of Representatives? Nancy Pelosi, Paul Ryan, Marco Rubio, John Boehner.

What job or political office is now held by Mike Pence? House Minority Leader, Vice President of the United States, Secretary of Defense, Secretary of State.

Whose responsibility is it to nominate judges to the U.S. Federal Courts? The President, the U.S. Senate, the U.S. House of Representatives, the Supreme Court.

How long is the term of office for a U.S. Senator? 2 years, 4 years, 6 years, 8 years.

Whose responsibility is it to determine if a law is constitutional or not? The President, the U.S. Senate, the U.S. House of Representatives, the Supreme Court.

How much of a majority is required for the U.S. Senate and House of Representatives to override a presidential veto? 1/2, 3/5, 2/3, 3/4.

Who is the current U.S. Secretary of State? Steve Bannon, Janet Napolitano, Rex Tillerson, Mike Pompeo.

Correct answers were coded 1; incorrect answers were coded 0. Responses to the 9 questions were averaged to form an index of political knowledge.

**Trust in Government**

How much of the time do you think you can trust the ***federal government*** in Washington, D.C. to do what is right? Almost always, most of the time, some of the time, almost never. Responses were coded to range from 0-1 with higher numbers meaning more trust.

**COVID Concern**

How concerned are you about a coronavirus outbreak in your local area? Extremely concerned, moderately concerned, a little concerned, not concerned at all, unsure. The first 4 response options were coded to range from 0-1, with higher numbers meaning more concern; “unsure” responses were treated as missing.

**COVID News**

How closely have you been following news about the outbreak of the coronavirus strain known as COVID-19? Very closely, fairly closely, not too closely, not at all closely. Responses were coded to range from 0-1, with higher numbers meaning more closely.

**Authoritarianism**

Although there are a number of qualities that people feel that children should have, every person thinks that some are more important than others. Below are pairs of desirable qualities. For each pair, please indicate which one you think is more important for a child to have.

Independence (coded 0), or Respect for elders (coded 1)

Curiosity (coded 0), or Good manners (coded 1)

Obedience (coded 1), or Self-reliance (coded 0)

Being considerate (coded 0), or Well behaved (coded 1)

Responses were averaged to form an index with higher numbers meaning more authoritarian.

**Party Identification**

Generally speaking, do you usually think of yourself as a *Democrat, a Republican, an Independent, or what*? Democrats and Republicans branched to: Would you call yourself a *strong Democrat [Republican]* or a *not very strong Democrat [Republican]*?  Independents and others branched to: Do you think of yourself as *closer to the Democratic Party* or *closer to the Republican Party*?

A Republican dummy variable was created such that 1 = Republican or lean Republican and 0 = Democrat/lean Democrat and pure Independents. An Independent dummy variable was created such that 1 = pure Independents and 0 = Republicans/leaners and Democrats/leaners.

**Learned Helplessness**

Responses to the 5 items were coded to range from 0-1 (with higher numbers meaning greater learned helplessness) and then averaged (alpha = .89).

Now we have a set of questions concerning how people feel. After reading each item, please respond as to how closely you agree or disagree with how each item describes you and your feelings about yourself. Strongly agree, agree, disagree, strongly disagree.

No matter how much energy I put into a task, I feel I have no control over the outcome.

Other people have more control over their success and/or failure than I do.

I feel that I have little control over the outcomes of my work.

I feel that anyone else could be better than me in most tasks.

No matter how hard I try, things never seem to work out the way I want them to.

**Personal Uncertainty**

Responses to the 3 items were coded to range from 0-1 (with higher numbers meaning more uncertainty) and then averaged (alpha=.87). Response options were: extremely uncertain, very uncertain, somewhat uncertain, not too uncertain, not uncertain at all.

At this very moment, how uncertain do you feel about yourself?

At this very moment, how uncertain do you feel about your place in the world?

At this very moment, how uncertain do you feel about your future?

**Conspiratorial Thinking**

Responses to the 4 items were coded to range from 0-1 (with higher numbers meaning more conspiratorial) and then averaged (alpha = .81).

How much do you agree or disagree with the following statements? Strongly agree, somewhat agree, neither agree nor disagree, somewhat disagree, strongly disagree.

Much of our lives are being controlled by plots hatched in secret places.

Even though we live in a democracy, a few people will always run things anyway.

The people who really 'run' the country are not known to the voters.

Big events like wars, economic recessions, and the outcomes of elections are controlled by small groups of people who are working in secret against the rest of us.
